# Unexpected shock in a fallen older adult: a case report

**DOI:** 10.29045/14784726.2020.06.5.1.15

**Published:** 2020-06-01

**Authors:** Gary Matthews, Helen Booth, Gregory Adam Whitley

**Affiliations:** East Midlands Ambulance Service NHS Trust; East Midlands Ambulance Service NHS Trust; East Midlands Ambulance Service NHS Trust

**Keywords:** accidental falls, emergency medical services, shock

## Abstract

**Introduction:**

Falls are common in older adults and frequently require ambulance service assistance. They are the most frequent cause of injury and associated morbidity and mortality in older adults. In recent years, the typical major trauma patient has changed from being young and male to being older in age, with falls of < 2 metres being the most common mechanism of injury. We present a case of an 84-year-old male who had fallen in his home. This case highlights the complex nature of a relatively common incident.

**Case presentation:**

The patient was laid on the floor in the prone position unable to move for 12 hours. He did not complain of any pain in his neck, back, hips or legs, and wished to be lifted off the floor promptly. On examination, he had bruising to his chest and abdomen and had suffered a suspected cervical spine injury due to a step-like protrusion around C5–C6. Distal sensory and motor function was intact. While in the ambulance his blood pressure dropped from 154/119 mmHg to 49/28 mmHg unexpectedly. We successfully reversed the shock using the modified Trendelenburg position and intravenous fluids. On follow-up he was diagnosed with dislocated C3, C6 and C7 vertebrae.

**Conclusion:**

The unexpected episode of shock witnessed in this patient may have been caused by a number of phenomena, including but not limited to crush syndrome, spinal cord concussion and orthostatic hypotension. We recommend that clinicians anticipate sudden shock in older adult patients who have fallen and a) have remained static on the floor for an extended period of time or b) are suspected of a spinal injury. We recommend assertive management of these patients to mitigate the impact of shock through postural positioning and consideration of early cannulation.

## Background

Falls are common in older adults (Darnell et al., 2012) and frequently require ambulance service assistance (Logan et al., 2010; Marks et al., 2002). They are the most frequent cause of injury and associated morbidity and mortality in older adults (McMahon et al., 2011). In recent years, the typical major trauma patient has changed from being young and male to being older in age, with falls of < 2 metres being the most common mechanism of injury (The Trauma Audit and Research Network, 2017).

We present a case of an 84-year-old male who had fallen in his own home and suffered an unexpected episode of shock in the ambulance prior to departure to hospital.

## Case presentation

### Initial assessment

The patient stated that he tripped and fell the previous night having consumed three glasses of whiskey, and had been on the floor for approximately 12 hours. We found the patient in the prone position, head facing right, both arms by his sides, head against the skirting board. Due to reduced mobility of his upper limbs caused by a previous injury, he was unable to move and had therefore been static in the same position for the full 12 hours.

We palpated his radial pulse, which was strong and regular. We then palpated his cervical spine, back, hips and legs for pain, which were negative. We assessed the sensory and motor function of his arms and legs, which were intact. We concluded from this initial assessment that injury to his cervical spine was unlikely.

Due to the extended time on the floor, we considered rolling the patient onto a scoop stretcher to mitigate any postural hypotension and to ease the burden on his potentially stiff joints; however, egress would not have been possible due to the narrow and convoluted hallway.

We rolled the patient onto his back and into a sitting position. He was able to bend both knees and raise both legs off the ground. We noticed a laceration approximately 2–3 inches to the top of his head which was not actively bleeding, and the patient’s head was in an unusual position, chin to his chest, head turned left (torticollis) which the family informed us was not normal. We proceeded to stand the patient to assess weight-bearing capability and to extricate from the confined space. The patient stood well, did not complain of dizziness or feeling light-headed and was without pain. We sat the patient on a carry chair and transported to the ambulance for a full assessment.

### Full assessment

We exposed and examined the patient in the ambulance, which revealed bruising/pooling along the anterior aspect of his torso, from the neck to the waist; this was non-painful to touch. The patient had been incontinent of urine. We inspected his cervical spine again due to the torticollis and noted deformity around C5 to C6 with a step-like protrusion. We palpated his cervical spine again which was negative for pain. Distal sensory and motor function was still intact. The patient denied chest pain, denied abdominal pain, was not short of breath and not complaining of any other pain.

#### Observations

A full set of observations were taken on the ambulance; see Table 1.

**Table 1.** Initial observations.

Observation	Reading	Units
Heart rate	90	beats per minute
Respiratory rate	20	breaths per minute
Blood pressure	154/119	mmHg
Oxygen saturation	98	% (on air)
Temperature	36.2	^o^C
Blood sugar	7.1	mmol/L
Glasgow Coma Scale	15	
Pain score*	0	

*Numeric pain rating scale.

### Management

Due to the deformed cervical spine, we wanted to apply a cervical collar and immobilise the patient on a scoop stretcher with headblocks. This was not possible due to the torticollis. We were unwilling to attempt realignment of the head due to fear of worsening the injury; therefore, as the patient was comfortable and without pain, we allowed him to maintain his own neck position. We carefully applied a wet dressing and bandage to the laceration on top of the patient’s head.

#### Unanticipated event

The patient became less responsive during the assessment and his blood pressure dropped to 49/28 mmHg. During this episode, the patient’s heart rate remained within a normal range at 74 bpm, with his respiratory rate raised slightly to 24 bpm and his breathing more laboured.

We immediately laid the patient flat and raised his legs (modified Trendelenburg position). During this time the patient responded to voice but was very confused with sluggish speech. We gained bilateral intravenous access with 18-gauge cannulas and administered 500 ml sodium chloride as a stat dose. We were unable to gain an accurate oxygen saturation reading, so we applied 100% oxygen for the remainder of the treatment. The patient responded well to the postural manoeuvre, intravenous fluid and oxygen. Post-event 12-lead electrocardiogram showed atrial bigeminy.

We pre-alerted the local emergency department, which was also a trauma unit, and transported the patient under emergency conditions. On arrival at hospital his observations were: blood pressure 141/85 mmHg, heart rate 75 bpm, respiratory rate 20 bpm and a Glasgow Coma Scale score of 15.

## Follow-up and outcomes

On follow-up the next day, the patient had been admitted to the orthopaedic ward with dislocated C3, C6 and C7 vertebrae. He was awaiting MRI imaging. The ward staff were unaware of the episode of shock experienced in the ambulance.

## Discussion

### Cervical spine immobilisation

Having determined cervical spine injury unlikely on initial assessment and subsequently identifying torticollis after moving and deformity during the second in-depth assessment, we were unsure why the injury was not detected initially. Dislocation of the cervical spine can occur without pain, weakness or paraesthesia (Akhaddar & Boucetta, 2010). Therefore, the absence of these symptoms made the identification difficult. In addition to this, pre-hospital environments are unpredictable and consequently make the assessment of trauma patients more challenging for clinicians (Abelsson & Lindwall, 2012). Due to the positioning of this patient in a confined space with low-level lighting, the initial assessment was extremely difficult; therefore, careful extrication to the ambulance for a full assessment was the most appropriate and pragmatic solution.

Spinal immobilisation using rigid cervical collars and hard backboards has played a significant role in pre-hospital care since the mid-1960s, with its appropriateness being increasingly questioned in recent years (Maschmann et al., 2019). Due to the clear deformity noted to the patient’s cervical spine and because the patient could not rotate their head 45^o^ to the left and right (Association of Ambulance Chief Executives, 2019), we were confident that immobilisation was the correct treatment pathway; however, we were not able to perform this. Current UK Joint Royal Colleges Ambulance Liaison Committee (JRCALC) guidelines (Association of Ambulance Chief Executives, 2019) state that the use of spinal immobilisation devices may be difficult (due to deformity) and could be counterproductive (increase pain, worsen neurological symptoms). Therefore, we were reassured that our decision not to attempt immobilisation for pragmatic reasons was justified.

### Shock

The episode of shock was unexpected and we were unsure of the cause. During the management, we believed this response was a result of crush syndrome due to the prolonged time (12 hours) laid on the floor in the prone position without movement. We believed that the bruising/pooling evident on the patient’s torso was a sign of skeletal muscle damage due to the pressure, and that the subsequent release of ‘toxins’ from the injured site when we rolled the patient over caused a delayed episode of shock. However, having been diagnosed with cervical dislocations at C3, C6 and C7, we feel that spinal cord concussion may have contributed to the shock. Also considering the long downtime and potential for dehydration, orthostatic hypotension was considered a contributing factor.

#### Crush syndrome

Crush syndrome, also known as rhabdomyolysis, occurs when skeletal muscle has sustained significant damage from a prolonged crush (usually 4–6 hours or more) (Gonzalez, 2005), causing metabolic changes due to the release of contents from the site of injury into the circulation (Rajagopalan, 2010). Crush syndrome patients present normally at first release, but soon go into shock (Rajagopalan, 2010; Sever & Vanholder, 2011). According to Rajagopalan (2010), potassium is released into the circulation which causes alteration in cardiac rhythm. We noted abnormalities on the ECG (atrial bigeminy) which may indicate crush syndrome as a predominant cause of the shock.

##### Pre-hospital management of crush syndrome

Pre-hospital management of crush syndrome should focus on early aggressive hydration and forced diuresis (Gonzalez, 2005). According to UK JRCALC guidelines (Association of Ambulance Chief Executives, 2019), crushed limb injuries require 2 litres initial stat dose of sodium chloride, whereas crushed torso injuries require a titrated dose to maintain central pulses, up to 2 litres.

#### Spinal cord concussion

Spinal cord concussion is a variant of mild spinal cord injury that normally resolves over a period of 24–72 hours after injury (Del Bigio & Johnson, 1989; Fischer et al., 2016). It often occurs when vertebral abnormalities are pre-existing that narrow the spinal canal or create areas of hypermobility (Del Bigio & Johnson, 1989). The mechanism in which spinal cord concussion could cause shock is likely through deregulation of sympathetic outflow, as is common in cervical spinal cord injuries (Hou & Rabchevsky, 2014). This allows parasympathetic outflow to dominate, causing cardiovascular compromise. Spinal cord concussion is poorly understood and underreported, and little is known about its immediate consequences and long-term effects (Fischer et al., 2016).

The lack of neurological defect with this patient questions the severity of spinal cord injury, if any; therefore, we believe spinal cord concussion was more likely than neurogenic shock in this case.

##### What is neurogenic shock?

Neurogenic shock is rare and notoriously difficult to define. Spinal cord injury is considered pertinent to the definition, resulting in a loss of sympathetic output and a decrease in heart rate and blood pressure (Guly et al., 2008; Lloyd, 2016; Taylor et al., 2017).

##### Pre-hospital management of spinal cord concussion

Pre-hospital management of shock caused by spinal cord concussion is difficult to establish with any confidence due to the limited evidence. Considering the assumed similarity in mechanism to neurogenic shock, similar treatment recommendations should be followed. This includes maintaining tissue perfusion with judicious crystalloid administration (Lloyd, 2016) to achieve a systolic blood pressure > 90 mmHg (Association of Ambulance Chief Executives, 2019).

On reflection, it was fortunate that the perceived crush syndrome was caused by a torso injury as opposed to a limb injury, as the management of crushed torso injuries and spinal cord concussion are both similar in terms of fluid replacement (Association of Ambulance Chief Executives, 2019). If a crushed limb injury had been sustained, there would have been a conflict of treatment pathways and more senior clinical advice would have been sought.

#### Orthostatic hypotension

The mechanism of orthostatic hypotension, also called postural hypotension, is well understood and is characterised by a drop in blood pressure when standing after sitting or lying down (National Institute for Health and Care Excellence, 2013). It is more common in older people (Rutan et al., 1992) and is exacerbated by dehydration. This patient was lying on the floor for a significant amount of time, consumed three glasses of whiskey the night before and had consumed no other fluids in the previous 12 hours. It was likely he was becoming dehydrated. This coupled with the extensive time on the floor could have caused a degree of orthostatic hypotension. This may have contributed to the shock.

##### Pre-hospital management of orthostatic hypotension

Pre-hospital management of orthostatic hypotension often involves postural positioning, including the modified Trendelenburg position. If dehydration is considered a contributing factor (National Institute for Health and Care Excellence, 2013), then slow administration of sodium chloride up to 2 litres over many hours is recommended (Association of Ambulance Chief Executives, 2019).

## Conclusion

The unexpected episode of shock witnessed in this patient may have been caused by a number of phenomena, including but not limited to: crush syndrome, spinal cord concussion and orthostatic hypotension.

We recommend clinicians anticipate shock in older adult patients who have fallen and a) have remained static on the floor for an extended period of time or b) are suspected of a spinal injury. We recommend assertive management of these patients to mitigate the impact of shock through postural positioning and consideration of early cannulation.

## Take-home points

- Cervical dislocations can occur without pain or neurological deficit, so vigilance when assessing clinical signs such as deformity and torticollis is essential.
- Anticipate shock in fallen patients with suspected spinal injuries.
- Anticipate crush syndrome in fallen patients who have remained static on the floor for a significant period of time (4+ hours).
- Actively mitigate shock in these patients with postural manoeuvring where possible (lay flat) and early consideration of intravenous access.

## Acknowledgements

We thank the patient and his relatives for giving consent to publish this case report. We also thank Mr Roger Linnell and Dr Simon Topham for expert advice on this case.

## Author contributions

All authors attended this case. GAW drafted the initial manuscript. All authors contributed to the revision and agreed on the final version of the manuscript. GM acts as the guarantor for this article.

## Conflict of interest

None declared.

## Funding

None.

## Informed consent

We gained verbal consent from the patient and his relatives to publish this case report.
